# 

**Published:** 1902-06-21

**Authors:** 


### IMPORTANT NOTICE.

**On account of the Coronation Holidays, we beg to announce that we shall go to press with our issue of the 28th JUNE on MONDAY NIGHT, the 23rd JUNE. Advertisements intended for insertion in this issue should therefore reach us by 4 p.m. ON MONDAY, the 23rd JUNE.**

### NOTICE TO CORRESPONDENTS.

All MSS., Letters, Books for Review, and other matters intended for the Editor should be addressed THE EDITOR, "THE HOSPITAL," THE HOSPITAL BUILDING, 28 & 29 SOUTHAMPTON STREET, STRAND, W.C.

The Editor cannot undertake to return rejected MS., even when accompanied by stamped directed envelope.

**Notice to Advertisers and Subscribers.**

All Advertisements, Orders for Copies of THE HOSPITAL, and Business Communications should be addressed to **THE MANAGER, (Not to the Editor)**, THE HOSPITAL, 28 & 29 Southampton Street, Strand, London, W.C.

The Cost of Subscription, post free, is as follows.—Per week, 2½d.; per quarter, 2s. 8d.; per half-year, 5s. 3d.; per annum, 10s. 6d. Foreign:—Per annum, 15s. 2d., payable, in all cases, in advance.

**NOTICE.—Vol. XXXI. of "The Hospital" (October 1901, to March 1902), in handsome cloth binding is now on sale at the office of this Journal, price, 7s. 6d. Cases for binding the Numbers contained in this Volume 1s. 6d. Index to Vol. XXXI., 2½d., post free.**

**The Monthly Part for June is now ready. Price 6d., by post 8d.**

### THE STUDENT OF MEDICINE.

### APPOINTMENTS.

**Geo. Alex. Heaton Barton**, M.D.Brux., M.R.C.S., L.R.C.P., L.S.A.Lond., appointed Second Anæsthetist to the North-West London Hospital. **Harold Knowles Bean**, M.D., C.M.Edin., appointed Government Medical Officer and Vaccinator at Wallsend, New South Wales. **George Brown**, M.B., appointed Senior House-Surgeon to the Dunedin Hospital, New Zealand. **T. R. Beale Browne**, M.R.C.S., L.R.C.P., appointed Junior Assistant Medical Officer to the Northampton County Asylum. **G. Burnside Buchanan**, M.B., C.M., F.F.P.S.G., appointed Assistant Surgeon to the Western Infirmary, Glasgow. **T. Harrison Butler**, M.A., M.D.Oxon., M.R.C.S., L.R.C.P., appointed Clinical Assistant to the British Ophthalmic Hospital at Jerusalem. **Robert H. Cole**, M.B., LL.B.Melb., appointed Coroner for Victoria. **S. V. Duncan**, M.R.C.S, L.R.C.P.Lond., appointed Medical Officer and

Public Vaccinator for the District of Kookynie, Western Australia. **John Farrington**, M.R.C.S., L.R.C.P.Lond., appointed Provincial Medical Officer, Fiji. **Wilfrid Glegg**, M.D., C.M., M.R.C.P.Edin., appointed Honorary Assistant Surgeon to the Birmingham and Midland Ear and Throat Hospital. **Reginald Green**, M.D., B.S., D.Hy.Durh., M.O.H. Gateshead, C.B., appointed Medical Officer of Health and Medical Officer to Fever Hospitals of the King's Norton and Northfield Urban District Council. **E. J. Gurdon**, L.R.C.P.Edin., M.R.C.S.Eng., appointed Health Officer for Nannine, Western Australia. **F. W. Hewitt**, M.A., M.D.Cantab., appointed Physician-Anæsthetist to St. George's Hospital. **G. Aubrey Jelly**, F.R.C.S.Edin., L.R.C.P.Lond., appointed Assistant Surgical Officer of the Manchester Royal Infirmary. **W. E. Jones**, M.R.C.S., L.R.C.P., appointed Medical Superintendent to Brecon and Radnor Joint Counties Asylum. **William Lamb**, M.D., C.M.Edin., M.R.C.P.Lond., appointed Honorary Surgeon to the Birmingham and Midland Ear and Throat Hospital. **Wm. C. McCann**, L.R.C.P., L.R.C.S.Irel., appointed Medical Officer of the Longford Dispensary District. **William McGee**, L.K.Q.C.P.I., appointed Health Officer for Phillip Island and Woolamai, Victoria. **Charles N. Macquarie**, L.R.C.P., L.R.C.S.Edin., appointed Medical Officer of Health for the Shire of Omeo, and Public Vaccinator for the North-Eastern District, Victoria. **Archibald Mahomet**, M.B., Ch.B.Aberd., appointed House Surgeon to the Paddington Green Children's Hospital. **J. Noonan Meade**, appointed Medical Officer and Public Vaccinator for Lybster, Caithness. **F. Norton Menzies**, M.B., appointed House Physician to the Hospital for Sick Children, Great Ormond Street. **G. Victor Miller**, M.D., appointed Consulting Ophthalmic and Aural Surgeon to the Stockton and Thornaby Hospital. **J. R. Mitchell**, M.B.Durh., appointed District Medical Officer of the Tynemouth Union. **T. Gillman Moorhead**, M.B., B.Ch.T.C.D., L.M.Rotunda, appointed Assistant Physician to Sir Patrick Dun's Hospital, Dublin. **William E. Morgan**, M.R.C.S., L.R.C.P.Lond., appointed Medical Officer for the Camberwell District of the Camberwell Union. **Charles D. Musgrove**, M.D.Edin., appointed Medical Officer of Health for Penarth. **Daniel P. O'Brien**, F.R.C.S., L.R.C.P.Irel., appointed Visting Medical Officer to the Meteorpark Orphanage, Stanwell, Rockhampton, Queensland. **Edwin Stephen Pasmore**, M.D.Lond., appointed Medical Superintendent to the Croydon Borough Lunatic Asylum, Warlingham. **Thomas Shanasy**, L.R.C.P., L.R.C.S.Edin., appointed Health Officer for the Shire of Lowan, Victoria. **George Shorland**, M.R.C.S., L.R.C.P.Lond., appointed District Medical Officer of the Westbury Union. **A. Bannier Sloan**, Ch.B., M.D.Glasg., appointed Assistant Medical Officer to the Fever Hospitals of the Metropolitan Asylums Board. **Frederick H. Stewart**, B.A., M.B., B.Ch., B.A.O., appointed Fourth Assistant and Pathologist to the Kent County Lunatic Asylum, Barming Heath. **F. J. Stuart**, M.R.C.S., L.R.C.P. Lond., appointed Senior Assistant Medical Officer to the Northampton County Asylum. **Walter Sykes**, L.R.C.P.E., L.R.C.S.E., appointed Senior House Surgeon to the Birmingham and Midland Eye Hospital. **C. Thackray**, M.R.C.S., L.R.C.P.Lond., appointed First Assistant Medical Officer to the Bethnal Green Infirmary. **C. E. Todd**, M.D.Brux., L.R.C.P.Lond.. M.R.C.S.Eng., appointed Hon. Assistant Surgeon to the Adelaide Hospital, South Australia. **Alexander Trotter**, M.B., B.Ch Edin., appointed Resident Surgical Officer to the Birmingham and Midland Eye Hospital. **Thomas Henry E. Watts-Silvester**, M.R.C.S.Eng., L.R.C.P. Lond., appointed Assistant Medical Superintendent of the Brentford Union Infirmary. **G. T. Wilkinson**, L.R.C.P.Edin., L.R.C.P.I., appointed Medical Officer of Health to the Scalby District Council. **J. Wilkinson**, M.D.Edin., D.P.H., appointed Medical Officer of Health to the Droitwich Rural District Council. **B. M. Wilson**, M.B., B.Ch.Vict., appointed House Surgeon to the Manchester Royal Infirmary. **Geoffrey R. Wilson**, B.C.Camb., appointed House Physician to the Paddington Green Children's Hospital. **Sidney Worthington**, M.D.Lond., F.R.C.S.Eng., appointed H.M. Certifying Factory Surgeon for the Chesterfield District.

### "The Hospital" Institutional Library.

"Hospitals and Asylums of the World." 4 Vols., with a Portfolio of Plans, complete £12 12s.

"Burdett's Hospitals and Charities, 1902." 5s.

"Cottage Hospitals, General, Fever, and Convalescent." 10s. 6d.

"Hospital Expenditure: The Commissariat." 2s. 6d.

"A Legal Handbook for the Use of Hospital Authorities." 2s. 6d.

"The Cottage Hospital Case Book or Register of Patients." 10s. and 12s. 6d.

"The Furnishing and Appliances of a Cottage Hospital." 6d.

All these are published by the SCIENTIFIC PRESS, Ltd., and may be obtained through any bookseller, or direct from the publishers, 28 and 29 Southampton Street, Strand, London, W.C.

